# Tsv-N1: A Novel DNA Algal Virus that Infects *Tetraselmis striata*

**DOI:** 10.3390/v7072806

**Published:** 2015-07-17

**Authors:** António Pagarete, Théophile Grébert, Olga Stepanova, Ruth-Anne Sandaa, Gunnar Bratbak

**Affiliations:** 1 Department of Biology, University of Bergen, Bergen 5006, Norway; E-Mails: theophile.grebert@ens-lyon.org (T.G.); Ruth.Sandaa@uib.no (R.-A.S.); bratbak@uib.no (G.B.); 2 Département de Biologie, ENS de Lyon, Lyon 69007, France; 3 Institute of Natural and Technical Systems, Russian Academy of Sciences, Sevastopol 299000, Russia; E-Mail: solar-ua@ya.ru

**Keywords:** microalgae, viruses, viral genomics, viral taxonomy, infection dynamics, viral factory, horizontal gene transfer

## Abstract

Numbering in excess of 10 million per milliliter of water, it is now undisputed that aquatic viruses are one of the major factors shaping the ecology and evolution of Earth’s microbial world. Nonetheless, environmental viral diversity and roles remain poorly understood. Here we report the first thorough characterization of a virus (designated TsV) that infects the coastal marine microalga *Tetraselmis striata*. Unlike previously known microalgae-infecting viruses, TsV is a small (60 nm) DNA virus, with a 31 kb genome. From a range of eight different strains belonging to the *Chlamydomonadaceae* family, TsV was only able to infect *T. striata*. Gene expression dynamics revealed an up-regulation of viral transcripts already 1 h post-infection (p.i.). First clear signs of infection were observed 24 h p.i., with the appearance of viral factories inside the nucleus. TsV assembly was exclusively nuclear. TsV-N1 genome revealed very different from previously known algae viruses (*Phycodnaviridae*). Putative function and/or homology could be resolved for only 9 of the 33 ORFs encoded. Among those was a surprising DNA polymerase type Delta (only found in Eukaryotes), and two genes with closest homology to genes from human parasites of the urogenital tract. These results support the idea that the diversity of microalgae viruses goes far beyond the *Phycodnaviridae* family and leave the door open for future studies on implications of microalgae viruses for human health.

## 1. Introduction

The acknowledgement of the importance of viruses in the marine environment started in the late 1970s, with the first successful cultivation of a virus infecting a microalga (eukaryotic phytoplankton) and the first estimates of their extreme abundance [1,2]. Indeed, viruses are the most abundant biological entities in the ocean, amounting to an average of 10^7^ viruses per mL of seawater. They outnumber bacteria by 10 to 1, and are responsible for an estimated 10^23^ infections per second, killing about 20% of the oceans microbes on a daily basis [2,3,4]. In addition to being numerically relevant, phytoplankton viruses are of tremendous ecological, biogeochemical and evolutionary importance. Viral lysis diverts the flow of organic carbon passed up to higher levels of the food web toward heterotrophic microbes (a process called the viral shunt), but also stimulates primary production by releasing growth-limiting nutrients such as nitrogen and phosphorus [5]. These two processes in turn affect the efficiency of the biological carbon pump. The massive mortality also impacts population dynamics and community composition in the marine environment, as exemplified by the implication of viruses in the control and termination of various microalgal blooms (extensive proliferation of microalgae) [5,6].

Environmental viral diversity and roles, especially where links to human health and well-being are not obvious, remains nonetheless poorly understood. Metagenomic surveys in the marine environment commonly report very high levels of previously unseen viral-encoded genetic diversity [7,8]. Viruses potentially contribute to the existence of high planktonic diversity levels [3,9,10], and certainly play an important role in microbial evolution as major vectors of horizontal gene transfer (HGT) [3,6,11,12,13].

Viruses infecting eukaryotic phytoplankton usually show high levels of host specificity, infecting a single particular species if not particular strains within a species [1,14,15]. In contrast, the same algal strain can often be parasitized by different viruses, which can be totally unrelated. This observation, together with the abundance of algal viruses in the marine environment (and the observation that microalgae susceptible to viral infection include representatives from almost all algal phyla), suggests that every algal species is susceptible to at least one virus [1]. Interestingly, almost all known algal viruses are lytic, but some algae may acquire resistance to cell lysis, resulting in “chronic” formation of viral particles without cell death [1,9]. To date, about 50 eukaryotic-algae virus genomes have been fully sequenced. Most of those are large double-stranded DNA viruses, grouped in the family *Phycodnaviridae* (supergroup of the “nuclear-cytoplasmic large DNA viruses”, NCLDV), with genomes ranging from 160 to 560 kb and capsids ranging from 100 to 220 nm [1,14]. Some algae viruses harbor genes as unexpected as RNA polymerase, tRNAs or DNA repair proteins [7,13,14]. However, the exact extent of this diversity remains elusive, as the vast majority of these genes are of unknown function and seldom have matches in public databases [1,14]. Small DNA viruses infecting diatoms (genome size of 5.6 to 6 kb) or *Raphidophyceae* (38.5 kb) as well as RNA viruses have also been reported [14,16].

Here we report the first thorough characterization of a virus (here designated TsV) that infects *Tetraselmis striata* (*Chlorophyta*). *Tetraselmis* are large (6–10 μm) unicellular green algae members of the *Chlorodendrophyceae*. Cells are covered by a thin cell wall (theca) made of scales that are fusioned extracellularly. Their four equal flagella emerge from an anterior pit and are covered with hair, and they harbour a unique posterior chloroplast with an eyespot and a pyrenoid. Some species have been isolated from freshwater habitats, but *Tetraselmis* are usually considered halophilic. Some *Tetraselmis* have been found as endosymbionts of acoel flatworm or radiolarian [17,18,19,20]. The habitat of *Tetraselmis* in the marine environment is not well characterized, but species have been described from coastal temperate, temperate-cold and tropical waters [18,19,20]. *Tetraselmis* are commonly used to feed larvae in aquaculture, and potential applications are being investigated in energy production, extraction of compounds of interest or pollution bioremediation [17,21,22]. A number of viruses infecting *Tetraselmis* have earlier been isolated from the Black Sea [23]. In this study, we use TsV-N1 to characterize its host range, infection dynamics, genomic composition, and to investigate its presence in environmental metagenomic datasets.

## 2. Materials and Methods

TsV-N1 viral isolate was used in this study. This virus was isolated in February 2007 from Nordåsvannet, a very closed fjord branch in Bergen, Norway (60.33° N, 5.33° E), with a very low-salt (15 ppt) brackish surface layer. The virus lysate was obtained by adding 1 mL of 0.2 μm-filtered seawater to 50 mL exponentially-growing host *Tetraselmis* sp. strain UiB. Once clearing of the host culture was observed, the lysate was passed through a 0.2 μm syringe filter. A single virus clone, designated TsV-N1, was obtained by serial dilution to extinction three times. The highest dilution to lyse the host culture was passed through a 0.2 μm syringe filter for use in subsequent inoculations.

This isolate was used to study host range, infection dynamics, genomic composition, and to investigate its presence in environmental metagenomic datasets. Extensive methodological description can be found in the Supporting Information.

### 2.1. Testing Host Range

Besides the original *Tetraselmis* strain used to isolate the virus, host range infectivity was tested on 7 other *Chlorophyta* algae strains, belonging to the genera *Mantoniella*, *Micromonas*, *Pyramimonas*, *Pseudoscourfieldia*, and *Tetraselmis* (3 strains). For each infectivity test, 15 mL exponentially growing alga culture with concentration around 5 × 10^5^ cells·mL^−1^ were inoculated with 0.5 mL of viral lysate. Cell concentration was monitored every two days, using a Fuchs-Rosenthal counting chamber. Taxonomical classification of the tested microalgae was done based on 18S sequences, amplified from purified DNA with primers Euk1A [24] and Euk516r [25] (Table 1). Sequences were submitted to Genbank under the following ascension numbers: KT007548 to KT007555.

The 18S sequences of the 8 tested microalgae strains were blasted, using blastn 2.2.25 [26], against Genbank’s nt database to retrieve the nearest sequences known to date. 18S sequences for all known Tetraselmis species and other classes of algae were also retrieved from the SILVA rRNA database [27] to enhance resolution. A total of 38 18S sequences (including those of our 8 strains) where hence used in the phylogenetic reconstruction.

**Table 1:** List of primers used in this study.

Name	F/R	Sequence (5′-3′)	Targeted Gene	Hybridization Temperature for qPCR (°C)
Euk1a	F	CTGGTTGATCCTGCCAG	18S rDNA	n/a
Euk516r	R	ACCAGACTTGCCCTCC		
CDP_F339	F	AAACATCACCGTGCCAACAC	TsV_14 Putative Capsid Decoration Protein	58
CDP_R450	R	ATGAGCTTTCTTCGCGTACC		
DNAPol_F83	F	ATAAGCCCGAGCCGAAAAAG	TsV_19 Putative DNA Polymerase	59
DNAPol_R203	R	AATCCGAGATTCAGCTCCAGTG		

### 2.2. Infection Dynamics

Two liters of exponentially-growing *T. striata*, strain UiB, were divided in two 1 L aliquots. One of the aliquots was inoculated with 2 mL of TsV-N1 viral isolate. Each of the two treatments was then divided in 3 biological replicates (300 mL per replicate). Initial cell concentration was 9.3 × 10^4^ cells·mL^−1^. The cultures were sampled at 0, 1, 3, 6, 12 and 24 h after viral addition for flow cytometry, gene expression, and electron microscopy. The on-line application Primer3 [28] was used to design primers that target qPCR-adequate regions in two TsV-N1 genes, TsV_019 and TsV_014, encoding for putative DNA Polymerase (DNA Pol) and Capsid Decoration Protein (CDP), respectively (Table 1). Please refer to the Supplementary Info for detailed description.

### 2.3. TsV-N1 Genomic Analysis

Total genomic DNA of the virus was TsV-N1 collected from fresh lysate using PFGE. Briefly, viruses were concentrated by ultracentrifugation for 2 h at 28,000 rpm at 10 °C. Two viral agarose plugs were prepared from the 200 mL viral concentrate for PFGE. The agarose plugs were run on a 1% w/v SeaKem GTG agarose gel in 1× TBE gel buffer using a BioRad DR-II CHEF Cell (Bio-Rad, Richmond, CA, USA) electrophoresis unit. The band of interest (31 kb) was excised and frozen at −80 °C. DNA was eluted from the PFGE agarose gel slices in 10,000 MWCO cellulose dialysis membranes by electrophoresis.

The viral genome was subjected to enzymatic digestion to determine its nature (double- or single-stranded DNA, or RNA). For DNA digestion, the virus-plugs, prepared as for PFGE, were submerged in reaction buffer (20 mM Tris–HCl pH 8.3 with 2 mM MgCl_2_) for 4 h (4 °C). The reaction buffer was removed and buffer containing 0.5 mL DNase (15 mg/mL, 20.1 units/mg, Sigma-Aldrich Company Ltd., Dorset, UK) was added. DNA was digested for 18 h at room temperature. To digest both single stranded and double-stranded RNA, the virus-plugs were placed in 0.01× SSC buffer (1× SSC: 0.15 M NaCl, 0.015 M Sodium citrate, pH 7.0) for 4 h. The buffer was exchanged with buffer containing 3 U RNaseA (Sigma) and the RNA was digested overnight at 37 °C. All plugs were run on PFGE as described above.

For genomic sequencing, further concentration of the DNA was performed using Vivaspin 500 columns (Milipore Corp., Billerica, MA, USA). Eluted DNA from these bands was amplified based on a linker-adaptor PCR method using the WGA1 and Genome Plex WGA re-amplification kit from Sigma (Sigma-Aldrich Company Ltd., St Louis, MO, USA). Six separate WGA reactions were run and pooled before further processing. The amplified products were purified using the GenElute PCR Clean-Up Kit (Sigma-Aldrich) and stored at −80 °C until sequencing. Pyrosequencing was performed by the Joint Genome Institute (Walnut Creek, CA, USA) using a Roche/454 GS FLX Titanium pyrophosphate sequencing platform (454 Life Sciences, Branford, CT, USA), under the Gordon and Betty Moore Marine Microbiology Initiative (Genbank accession code JF974319).

The 33 open reading frames (ORFs) encoded in the TsV-N1 have been submitted to Uniprot and can be retrieved with taxonomy accession code 756284. Translated predicted ORFs were compared to the NR GenBank database by BLASTp and PSI-BLAST programs [26,29]. Conserved protein domains were identified using the InterProScan 5 program and NCBI Conserved Domain Search [30,31]. In cases where significant homology (e-value ≤ 0.001) with a conserved domain was observed, the whole family of genes containing that domain was retrieved from the PFAM database for further analysis. Redundancy of each specific database was reduced using the EMBOSS [32] tool “skipredundant” with a maximum sequence similarity threshold of 70%. Alignments of TsV_N1 ORFs with the rest of the domain representatives were performed both using MSAProbs and Maff L-ins-I algorithms from PFAM and Jalview ([33,34], respectively). After a manual curation of the alignments to remove potential artifacts, phylogenetic reconstruction for each gene family was attempted using the Maximum Likelihood aLRT SH-like algorithm implemented in Phylogeny.fr [35].

### 2.4. Environmental Metagenomic Analysis

The metagenomic environmental dataset from the Sorcerer II “Global Ocean Sampling Expedition” [36] was screened for the presence of TsV-N1 or *T. striata* reads. The viral size fraction (<0.1 μm) was available for only 4 sampling stations located in the Indian Ocean [8], representing different ecosystems: lagoon reef, open ocean, coastal waters, and open ocean. We used tBLASTx [26] with a maximal E-value of 10^−5^ to recruit reads similar to TsV-N1. BLASTn [26] was used with a maximum E-value of 10^−10^ to recruit *T. striata* 18S in the 0.1–0.8 μm and 0.8–3.0 μm size fractions. Positive hits were aligned with the 38 pre-aligned sequences previously used in the host range phylogenetic reconstruction (see above). Phylogenetic reconstruction was performed with PhyML under a GTR+I+G model, with 100 bootstrap replicates [37].

## 3. Results

### 3.1. Host Range

**Figure 1:**
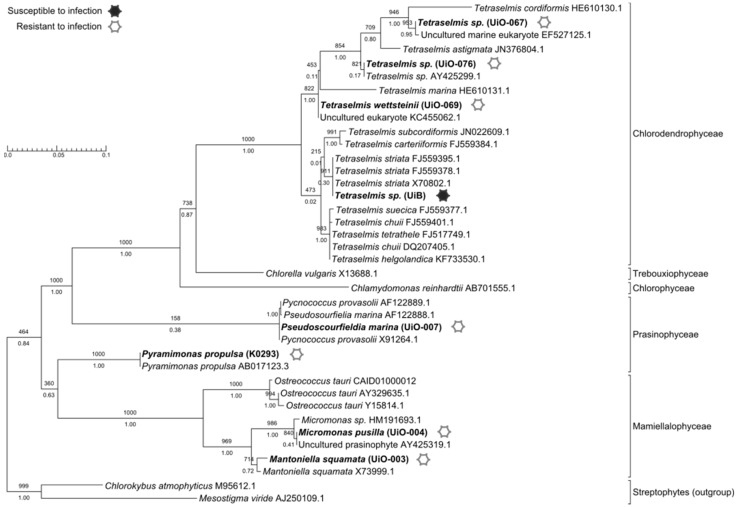
Host phylogeny and infectivity range of TsV-N1. The tree corresponds to the Maximum Likelihood phylogeny based on 18S sequences of 38 microalgae strains with 1000 bootstrap replicates (bootstrap values indicated above each branch). Bayesian posterior probabilities are indicated below each branch. The susceptible host strain is indicated with a filled symbol, while the other tested/resistant strains have an open symbol. Culture collection reference for the microalgae strains tested for viral infection is indicated between brackets. GenBank accession number is indicated after their name for sequences retrieved from public databases.

Host infectivity range was tested on eight microalgae strains of five different genera of *Chlorophyta*. The original host used to isolate the virus revealed to be a *Tetraselmis* *striata* strain, and was the only susceptible alga among the eight tested (Figure 1).

### 3.2. Infection Dynamics

**Figure 2:**
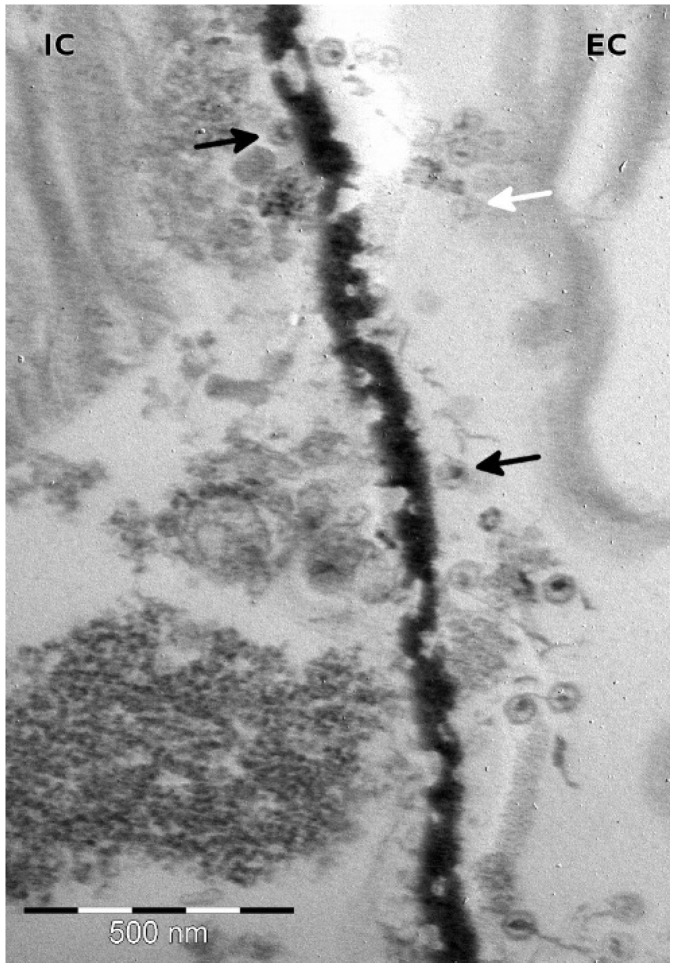
Thin section of a *T. striata* showing TsV-N1 particles inside (IC) and outside (EC) the cell. Black arrow: filled viral particle, white arrow: empty capsid. Note the hexagonal section of the virions.

The TsV-N1 particles presented a mean diameter of 60 nm (min 49 nm, max 73 nm, s.d. 5 nm, as measured on 100 full viral particles). They are tailless, and their sections appeared to be hexagonal (Figure 2).

**Figure 3:**
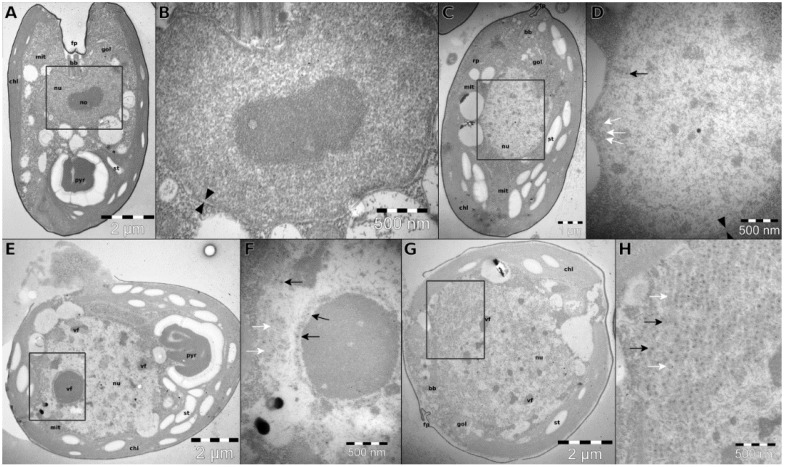
Ultrastructural changes in *T. striata* associated with the progression of TsV-N1 infection. (**A**) longitudinal section of an uninfected cell, with anterior flagellar pit and posterior pyrenoid; (**B**) nucleus of an uninfected cell, view corresponding to the frame in (**A**); (**C**) longitudinal section of an infected cell, first noticeable changes; (**D**) detail of the nucleus corresponding to the frame in (**C**); first viral capsids, intact nuclear envelope. (**E**) Longitudinal section of an infected cell with highly disordered nucleus; (**F**) enlargement of frame in (**E**) showing a viral factory (vf) inside the nucleus; (**G**) longitudinal section of a cell at the final stage of infection; (**H**) enlargement of frame in (**G**) showing numerous viral particles. Facing black arrowheads (**B**,**D**) nuclear envelope. Black and white arrows (**D**,**F**,**H**) full and empty viral particles. Legend: bb, basal bodies; chl, chloroplast; fp, flagellar pit; gol, golgi apparatus; mit, mitochondrion; no, nucleole; nu, nucleus; pyr, pyrenoid; rp, rhizoplast; st, starch grain; and vf, viral factory.

The first intracellular viral particles were observed in the nucleus 24 h p.i. and coincided with visible ultrastructural changes (Figure 3). Different stages of infection were recognized: first, the nucleus was disorganized, and mainly empty viral capsids (if any) were observed (Figure 3C,D). The nuclear envelope was still intact. During the second stage of infection (Figure 3E,F), a viral factory (electron dense structures surrounded by viral particles) could be seen in the nucleus. The nuclear envelope could not be observed at this stage, but the location matched the location of the nucleus in healthy cells (Figure 3A,B). Most capsids were full. In the last stage of infection (Figure 3G,H), the nuclear compartment was not recognizable anymore, the cytoplasm as well as the nuclear area were full with viral particles. Generally, only filled capsids could be observed.

Up to 800 particles were observed in one cell section (Figure 3G). Assuming the viral particle and the cytoplasmic region containing viruses to be spheres with respective diameters of 60 nm and 5 μm, and assuming the thickness of the TEM thin section to be 75 nm, a tentative burst size could be estimated around 4 × 10^4^ viruses per cell.

**Figure 4:**
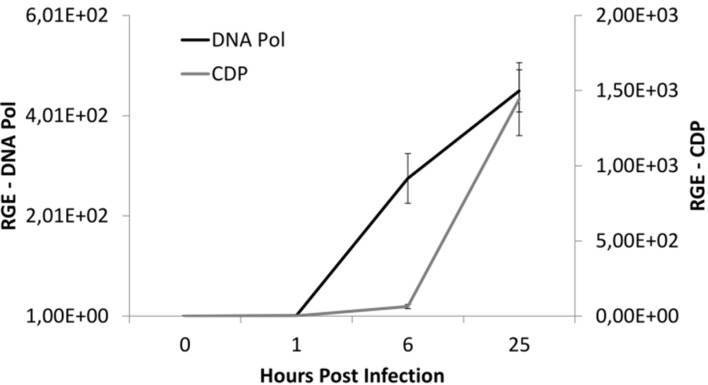
Relative expression of TsV genes TsV_019 and TsV_014, encoding for putative DNA Polymerase (DNA Pol) and Capsid Decoration Protein (CDP), respectively. RGE, relative gene expression units.

Gene expression measurements revealed increase in DNA Pol expression around 1 h p.i. CDP transcript numbers increased later, around 6 h p.i. (Figure 4). Relative increase of CDP expression during infection was higher than for DNA Pol. Although culture clearance due to cell lysis was clearly observed 5 days p.i., no significant difference was observed in cell concentrations between infected and non-infected cultures over the first 24 h p.i. (Figure S1).

### 3.3. TsV-N1 Genomic Analysis

Results from the enzymatic digestions showed that TsV-N1 has a dsDNA genome (Figure S2). High-throughput sequencing of the TsV-N1 genome resulted in three un-ordered contigs of 14,360 bp, 6886 bp, and 5305 bp, respectively, which amount to a total of 26,551 bp. This would correspond to *circa* 86% of the 31 kb total genome size estimated with PFGE.

Putative functions could be attributed to 8 out of the 33 (24%) genes encoded in the TsV-N1 genome (Table 2). We did not obtain any significant hit on a major capsid protein gene. However, a remote hit (e = 1E^−2^) was retrieved for Capsid Decoration Protein (TsV_14). Overall were identified genes involved in the different stages of viral replication machinery: transcription (TsV_08 and TsV_18), DNA replication (TsV_19), and capsid assembly (TsV_14). A rare type of DNA Polymerase, delta, previously known only to Eukaryotes, was found in the TsV genome (TsV_19).

**Figure 5:**
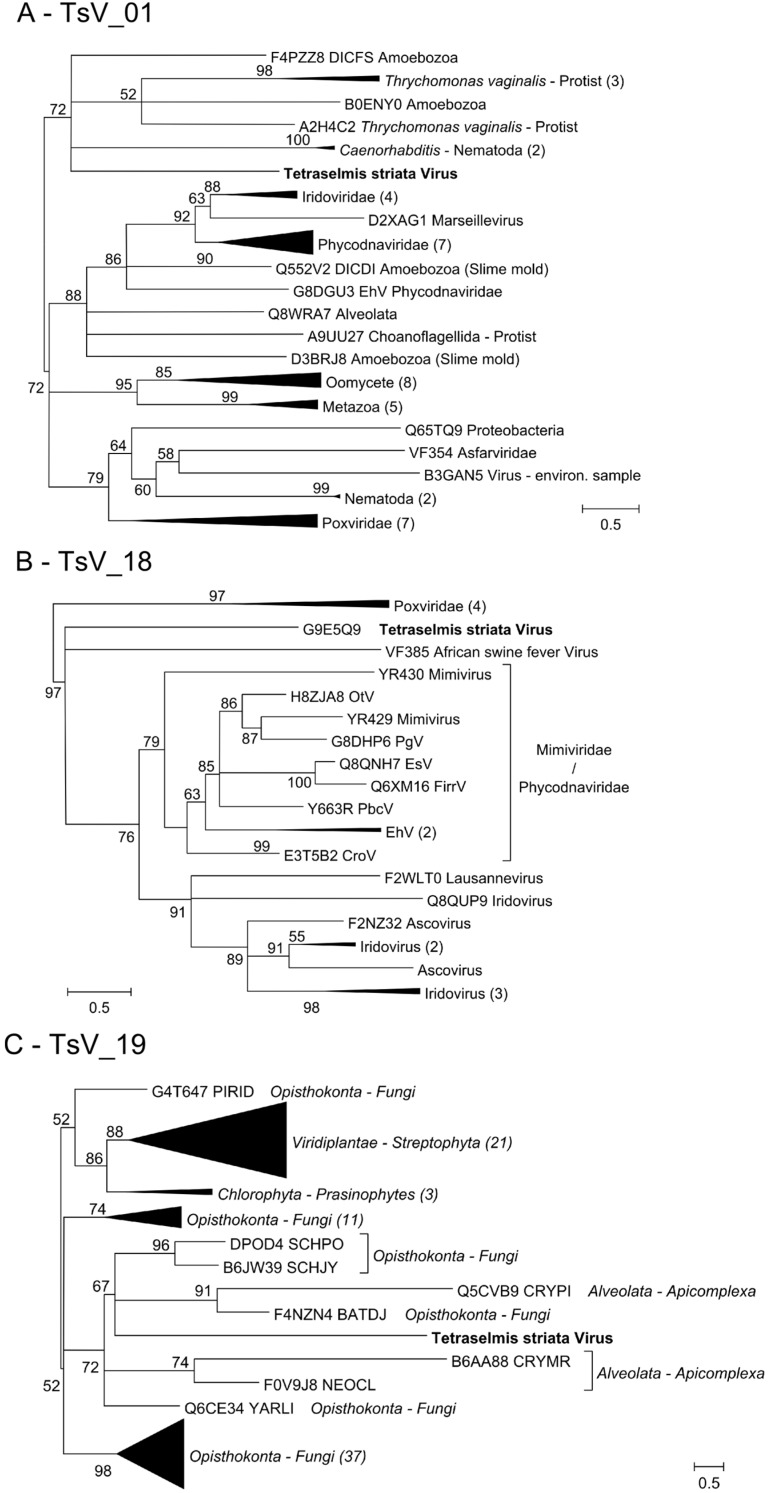
Phylogenies obtained for TsV_01, TsV_18, and TsV_19 genes, respectively, accompanied by homologous sequences retrieved from PFAM. Branches indicate support values obtained with Maximum Likelihood aLRT SH-like algorithm implemented in Phylogeny.fr.

In general, TsV sequences presented high levels of divergence regarding previously known genetic diversity. Consistent phylogenetic reconstruction was possible only for three TsV genes: TsV_01, TsV_18, and TsV_19 (Figure 5A–C, respectively). These genes were clearly placed outside the monophyletic phycodnavirus clades that were formed. Moreover, TsV_01 and TsV_19 revealed unexpected homologies with a eukaryotic human pathogen (*Trichomonas vaginalis*) (18.25%), and fungi (35.21%), respectively. Significant levels of TsV sequence similarity with unexpected cellular/viral domains were also observed for the other six genes (Table 2). Surprisingly, from the nine TsV genes analyzed, five shared closest homology/sequence similarity with Eukaryotic genes, three with viral genes (2 being phage), and one with bacteria.

Genes TsV_01 and TsV_08 presented unexpected similarity with genes from parasites of the human urogenital system, *T. vaginalis* and *Candida albicans*, respectively.

### 3.4. Detection of TV-N1 and Tetraselmis striata Reads in Environmental Datasets

Four stations of the GOS Expedition metagenome were screened for the presence of both TsV-N1 and *T. striata*. Between 514 (station GS122) and 1761 (station GS108) reads were retrieved when blasting the environmental reads against the genome of TsV-N1. The minimal observed E-value was between 10–16 and 10–24 for each station, corresponding to alignments of 60–65 nt and identities of 40.0%–56.9%. However, all the reads aligned on less than 23% of their length, and failed to pass our filtering process. Thus, no read corresponding to the genome of TsV-N1 was considered detected.

The same happened regarding the host. Between one and seven reads, depending on the station, matched the imposed criteria of E-value, identity and alignment length for the 0.8–3.0 μm size fraction. They exhibited different phylogenetic assignation, but none of them corresponded to *Tetraselmis*.

**Table 2:** Putative annotation as well as phylogenetic homology recovered for TsV-N1 genes.

Gene	Conserved Domains	Pfam	Consistent Phylogeny?	Monophyletic Phycodnavirus	Closest Homology	Psi-Blast Top Hit		
						Top Hit	E-value	Taxonomy
TsV_01	Poxvirus A32 protein	PF04665	yes	yes	*Trichomonas vaginalis* Eukaryota	—	—	—
TsV_18	Poxvirus Late Transcription Factor VLTF3 like	PF04947	yes	yes	Eukaryotic Virus	—	—	
TsV_19	DNA polymerase delta, subunit 4	PF04081	yes	yes	Fungi-Eukaryota	—	—	—
TsV_04	Apolipoprotein L	PF05461	no	—	Mammalia	*Microtus ochrogaster*	2.0 × 10^−11^	Eukaryota-Mammalia
TsV_07	GP16	—	no	—	—	Mycobacterium Phage Marcell	1.1 × 10^−2^	Virus-Caudovirales
TsV_08	ATP-dependent DNA helicase PIF1	PF13604	no	—	—	*Candida albicans*	1.2 × 10^−2^	Eukaryota-Fungi
TsV_010	—	—	no	—	—	*Chlorella variabillis*	1.0 × 10^−3^	Eukaryota-Chlorophyta
TsV_14	Capsid decoration protein	PF02924	no	—	—	Prochlorococcus phage P-SSM4	1.0 × 10^−2^	Virus-Caudovirales
TsV_25	Coagulation factor 5/8 C-terminal type domain	PF00754	no	—	—	*Pectobacterium carotovorum*	9.0 × 10^−8^	Bacteria Enterobacteriaceae

## 4. Discussion

Here we presented the first thorough analysis of TsV-N1, a new virus that infects the microalga *Tetraselmis striata*. The isolation of this virus had been reported [38] as TvV (infecting *Tetraselmis viridis*) but genetic characterization of the host (presented here) has identified it as *T. striata*. In accordance with this we here rename the virus strain TsV-N1 *Tetraselmis striata* Virus. To our knowledge, this is the first virus known to infect a member of the class *Chlorodendrophyceae*. The fact that at this stage we can still isolate the first virus to infect a member of a diverse microalgae class as is *Chlorodendrophyceae* (46 assigned species, ALGAEBASE) reinforces our current ignorance of the vast viral diversity existing in the marine environment [39]. A combination of infectivity essays with taxonomical characterization of potential hosts revealed the incapacity of TsV-N1 to infect other species among a range of *Chlorophyta* species (Figure 1). This result is potentially in accordance with the traditional idea of phytoplankton viruses being species-specific. Cross-species infectivity has so far been rarely observed in marine eukaryote viruses. Nonetheless, a few reports exist, namely for viruses of brown algae, the green algae *Ostreococcus* [1,40], and haptophytes [41]. The capacity to cross species borders and infect more than one host species might be more widespread than previously expected [41], and our results do not exclude the possibility of TsV-N1 being capable of infecting other cellular types (see further discussion below).

The latent period of TsV-N1 infection, according to TEM imaging, is comprised between 1 and 24 h. This result is in accordance with values for other marine viruses [16,42]. While there was a strong increase in TsV-N1 DNA Pol transcripts as early as 1 h p.i., estimates of cell concentration did not show a pronounced cell loss during the first 24 h after viral addition, even though there was a significant difference in cell concentration between healthy and infected cultures. This is reasonable since the effects on cell concentration may not be perceived during the initial latent period, even if the viral machinery is already fully functioning to kidnap the cell. As an example, Baudoux *et al.* [42] observed a decrease in host cell concentration after 36 h for viruses of another alga, *Phaeocystis globosa*, that have a latent period of about 10 h. While viral-induced lysis was clearly observed in *T. striata* cultures after five days of infection, a clear TsV-N1 population could not be distinguished using FCM, probably due to their small size. Thin section EM revealed canonical ultrastructural changes, with production of viral particles associated with viral factories located inside the nucleus [16,42]. A tentative burst size was estimated at around 4 × 10^4^ viral particles per cell. This value is relatively high, but not unusual for small viruses [1]. This high estimate of burst size for TsV-N1, which remains preliminary and hence should be taken with caution, is rather contradictory with the long time it takes for clear culture lysis to be observed (four to five days). This contradiction could eventually be explained by a low viability rate for this virus on this host, which again would lead to questions on the possibility of existing other cellular types TsV-N1 can infect with more success.

The doubts arising on the “true” host for TsV-N1 originate from its very distinct traits when compared to other known algae viruses. The *Phycodnaviridae* family is a highly diverse family of algae-infecting DNA viruses [43]. The most obvious common trait shared by all Phycodnaviruses are their large sizes, both in terms of capsid and genome (encoding hundreds of genes), which have granted them direct entry into the exclusive world of giant viruses [44,45]. Despite their obvious diversity and deep roots in the tree of life, Phycodnaviruses consistently appear as a monophyletic group within the vast nucleo-cytoplasmic large DNA viruses [46,47]. The study presented here has, however, clearly showed that TsV-N1 is distinct from the *Phycodnaviridae* virus family. TsV-N1 diameter is 60 nm (Figure 2), smaller than the smallest phycodnavirus (100 nm in diameter), its burst size is at least one order of magnitude bigger, and it is only assembled in the nucleus (Figure 3). The TsV-N1 nuclear assembly resembles what is observed for the virus HaNIV, which infects *Heterosigma akashiwo* (*Raphidophyceae*) [16]. As TsV-N1, HaNIV is produced in the nucleus. This virus is smaller than TsV-N1 (30 nm), but has a slightly bigger double stranded DNA genome of 38.5 kb. Unfortunately, the genome of this virus has not been fully sequenced. Preliminary results on partial sequencing suggest that HaNIV is also highly divergent from other known viruses [48], but in the absence of a published genome, the eventual relationship of TsV-N1 and HaNIV cannot be ruled out.

At this stage, what makes TsV-N1 really distinct is its genome: encoding “only” 33 ORFs, it is much smaller than those found in Phycodnaviruses. Moreover, phylogenetic reconstructions for three of TsV-N1 genes did not place those proteins either inside or in close vicinity to the Phycodnavirus clades (Figure 5). The TsV-N1 genome is very divergent from what is currently known, resulting in that only nine (27%) of its ORFs had any clear homology signal. Its analysis revealed a surprisingly cosmopolitan assortment of genes, sharing homologies among the different cellular domains, eukaryote, prokaryote, and viral, respectively. Most of these (5) presented closer homology with eukaryotic genes, and only one of those is algal (TsV_10). Homologies with other viral genes were unexpectedly scarce. Cases where TsV genes show a phylogenetic placement inside Eukaryotic clades, like happened for TsV_01 and TsV_19, can potentially result from horizontal gene transfers between viral and cellular world.

Here we should note that the closest homologues to those two TsV genes are found in parasites of the human urogenital system, *Trichomonas vaginalis* and *Candida albicans* (Figure 5A,C). A plausible explanation could be that we are again witnessing the result of horizontal gene transfers between viral and cellular worlds. In particular, these pathogens are among the main causes of vaginitis, a gynecological disease that causes itching, pain, and redness of the vaginal mucosa, often accompanied by vaginal discharge [49]. Viruses infecting another microalga, *Phaeodactylum tricornutum*, have been isolated from women’s cervical secretion samples that had previously been diagnosed with vaginitis [50]. Those women had been bathing in Black Sea beaches 2–5 months before the symptoms emerged. Adding to this complex puzzle, other strains of TsV have been isolated from water samples from those same locations. The TsV-N1 strain characterized here was also isolated from very anthropogenized coastal water. These remarkable coincidences deserve further investigation. They add up to the recent first three findings of possible human/pathogen/aquatic-virus interactions [50,51,52], probably the tip of an iceberg that we are just starting to uncover.

### Perspectives and Implications

The discovery of TsV, the first reported virus that infects *Chlorodendrophyceae*, emphasizes our current level of ignorance regarding viral diversity in aquatic environments, in particular for viruses other than *Phycodnaviridae*. Isolation and characterization of other viral strains infecting *T. striata*, is an obvious next step to shed a light on the evolution of TsV. The biogeographical extent of such viruses still has to be explored, as well as their ecological relevance, with particular attention to their potential interactions with human pathogens and health.

## Supplementary Files

Supplementary File 1
