# Supplementary material for: Tsv-N1: A Novel DNA Algal Virus that Infects Tetraselmis striata
Source: Viruses. 2015 Jul 17;7(7):3937–53. doi: 10.3390/v7072806 (PMC4517135; doi:10.3390/v7072806)
Supplement: Supplementary File 1 [file viruses-07-02806-s001.pdf]

Supplementary Materials

Materials and Methods

Genomic DNA Isolation and Sequencing

Total genomic DNA of the virus TsV-N1 was collected from fresh lysate using PFGE. Viruses in 34 mL of viral lysate were further concentrated by ultracentrifugation (Beckman L8-M with SW-28 rotor) for 2 h at 28,000 rpm at 10 °C. The viral pellet was dissolved in 200 mL of SM buffer (0.1 M NaCl, 8 mM $MgSO_4 \cdot 7H_2O$, 50 mM Tris-HCl, 0.005% (*w/v*) glycerin). Two viral agarose plugs were prepared from the 200 mL viral concentrate for PFGE. Lysis of the viral particles was performed in freshly made lysis buffer (250 mM EDTA pH 8.0, 1% SDS, 1 mg/mL Proteinase K). The agarose plugs were run on a 1% *w/v* SeaKem GTG agarose (FMC, Rockland, ME, USA) gel in 1× TBE gel buffer using a BioRad DR-II CHEF Cell (Bio-Rad, Richmond, Ca, USA) electrophoresis unit. The band of interest (31 kb) was excised and frozen at −80 °C.

DNA was eluted from the PFGE agarose gel slices in 10,000 MWCO Spectra/Por, Regenerated Cellulose dialysis membranes (Spectrum Laboratories Inc., Los Angeles, CA, USA) by electrophoresis in 1× TAE buffer (40 mM Tris-HCl, 1 mM EDTA, 40 mM acetic acid, pH 8.0) for 3 h at 70 V. Further concentration of the DNA was performed using Vivaspin 500 columns (Milipore Corp., Billerica, MA, USA) according to the manufacturer's protocol. Eluted DNA from these bands was amplified based on a linker-adaptor PCR method using the WGA1 and Genome Plex WGA reamplification kit from Sigma (Sigma Aldrich, St Louis, MO, USA). Six separate WGA reactions were run and pooled before further processing. The amplified products were purified using the GenElute PCR Clean-Up Kit (Sigma Aldrich) and stored at −80 °C until sequencing. Pyrosequencing was performed by the Joint Genome Institute (Walnut Creek, CA, USA) using a Roche/454 GS FLX Titanium pyrophosphate sequencing platform (454 Life Sciences, Branford, CT, USA), under the Gordon and Betty Moore Marine Microbiology Initiative (Genbank accession code JF974319).

Testing Host Range

Chlorophyta algae strains from the Culture Collection of the University of Oslo had been previously anatomically identified as belonging to the genera *Mantoniella* (UiO003), *Micromonas* (UiO004), *Pyramimonas* (UiO293), *Pseudoscourfieldia* (UiO007), and *Tetraselmis* (UiO067, 069, and 076). Algae strains were cultured in IMR/2 medium at 17 °C. Taxonomical re-classification of the tested microalgae was done based on 18S sequences. Briefly, 2 mL of non-infected culture were centrifuged at 14,000 g for 30 sec. DNA was extracted from the pelleted cells with the UltraClean Soil DNA Isolation Kit (Mo Bio Laboratories Inc., Carlsbad, CA, USA), according to manufacturer's instructions for cells hard to lyse. Quantity and purity of each DNA sample was measured with a NanoDrop ND1000 (NanoDrop Technologies, Wilmington, DE, USA). The 18S ribosomal gene was amplified from purified DNA with primers Euk1A [1] and Euk516r [2] (Table 1). DNA amplification was conducted using TaKaRa Ex Taq polymerase with the following program: initial denaturation with 3 min at 95 °C, 30 cycles with 30 sec at 94 °C, 45 sec at 55 °C, 60 sec at 72 °C, and a final elongation step of 5 min at 72 °C. PCR products were cleaned with DNA Clean & Concentrator-5 Kit (Zymo Research Corp., Freiburg, Germany). DNA labelling was made using BigDye Terminator (Life Technologies, Paisley, UK) with the following

program: 5 min at 96 °C, followed by 25 cycles of 96 °C for 5 sec, 50 °C for 5 sec, 60 °C for 5 min. For each strain, both forward and reverse strands were sequenced, using either Euk1A or Euk516r primers, respectively.

A total of 38 18S sequences (including those of our 8 strains) where hence used in the phylogenetic reconstruction. Sequences were aligned using MUSCLE 3.8.31 [3] using default parameters. The alignment was processed with jModelTest 2.1.4 [4] to select the best-fit model for ML tree reconstruction. The model selected by the Akaike Information Criterion was the GTR+I+G model. Phylogenetic inference was performed using this model as implemented in PhyML 20120412 [5], with 1000 bootstrap replicates, initial tree being found with BioNJ algorithm, and tree topology searched using the best of NNI and SPR. Base frequencies and substitution parameters were estimated by PhyML. Bayesian phylogenetic analysis were performed using MrBayes 3.2.1 [6] and the GTR+I+G model, priors and parameters being left to their default values. The analysis was run for 60×10^6 generations on 4 runs, with sampling every 10000 generations, resulting in 6001 samples. The output was checked with Tracer v1.6 [7], and the burn-in was set to 1001 (representing the first 10×10^6 generations). Posterior probabilities of the ML tree were calculated with the DendroPy Phylogenetic Computing Library v3.12.0 [8].

Infection Dynamics

2 L of exponentially-growing *T. striata*, strain UiB, were divided in two 1 L aliquots. One of the aliquots was inoculated with 2 mL of TsV-N1 viral isolate. Each of the two treatments was then divided in 3 biological replicates (300 mL per replicate). Initial cell concentration was 9.3×10^4 cells mL^{-1}. The cultures were sampled at 0, 1, 3, 6, 12 and 24 h after viral addition for gene expression, flow cytometry, and electron microscopy.

For flow cytometry, 1 mL of culture was sampled at each time point. Samples were fixed with 40 µL 25% glutaraldehyde and incubated at 4 °C for 30 min before being frozen in liquid nitrogen and stored at −80 °C until analysis. Cell concentrations were measured using a FACSCalibur flow-cytometer (Becton Dickinson, Franklin Lakes, NJ, USA).

For RNA extraction 15 mL of culture were sampled at each time point and centrifuged for 10 min at 7000 g (4 °C). The pellet was re-suspended in 250 µL of culture medium, frozen in liquid nitrogen and stored at −80 °C until extraction. RNA was extracted with the RNEasy Plant Mini Kit (Qiagen, Venlo, Netherlands). The protocol was adapted from the Plant Mini Kit protocol and involved a supplementary proteinase K digestion after the cell lysate filtering on QIAShredder spin column (Qiagen, Venlo, Netherlands): after this filtering step, 2 volumes of RNAse-free water were added to the sample. EDTA was added at a final concentration of 20 mM. Proteinase K was then added in two consecutive steps, at a final concentration of 100 µg/mL, with 10 min incubation time at 37 °C, each. RNA purity and concentration were assessed with a Nanodrop ND1000 spectrophotometer (Thermo Scientific, Waltham, MA, USA). Reverse transcription (RT) was performed on 200 ng RNA using the BioRad iScript cDNA Synthesis Kit. The on-line application Primer3 [9] was used to design primers that target qPCR-adequate regions in two TV-N1 genes, encoding for putative DNA Polymerase and Major Capsid Protein, respectively (Table 1). Real-time PCRs were performed on 1 µL of RT products (10 ng cDNA) with the BioRad iTaq Universal SYBR Green Supermix, according to the manufacturer's instructions. A C1000 Thermocycler (BioRad) was used with the following program: activation (30 sec at 95 °C), 36 cycles of amplification (5 sec at 95 °C, followed by annealing-extension for 30 sec at 58 °C and 59 °C, for MCP

and DNAPol, respectively, Table 1). For each sampling point, two technical replicates corresponding to two independent qPCR reactions were performed for each RT product, as well as one non-RT sample for DNA contamination control.

For transmitting electron microscopy 40 mL of culture were sampled at each time point and centrifuged for 10 min at 7.000 g (4 °C). The pellet was fixed with 25% glutaraldehyde and stored at 4 °C until further processing. Briefly, samples were negatively stained using osmium tetroxide, followed by uranyl acetate and lead citrate post-staining. Micrographs were acquired with a JEOL 1011 transmission microscope (JEOL, Tokyo, Japan), with an accelerating voltage of 100 kV.

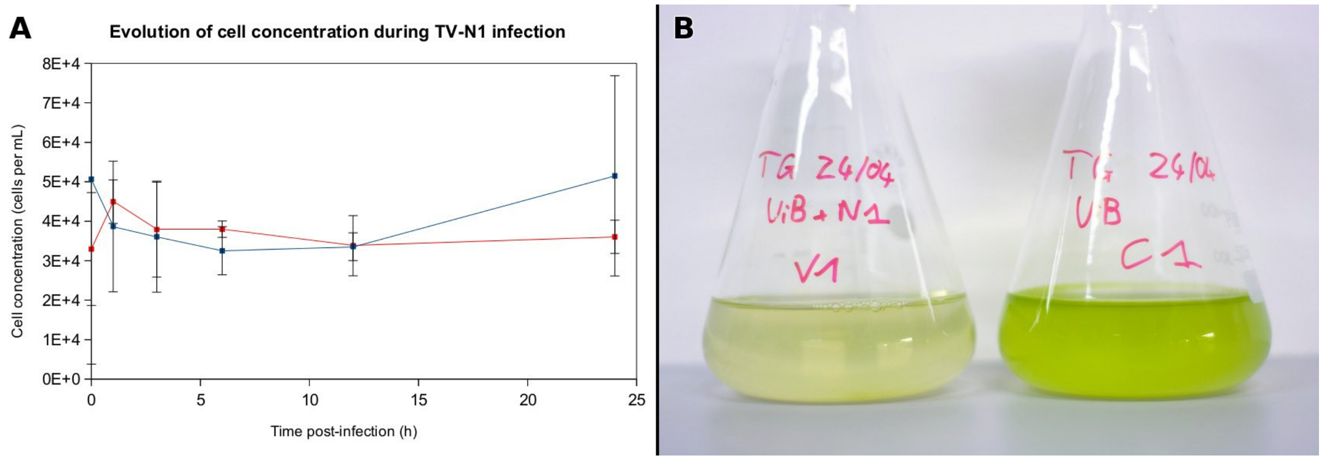

Figure S1. Evolution of cell concentration during TsV-N1 infection. (**A**) Cell concentration estimated by flow-cytometry at 0, 1, 3, 6, 12 and 24 h after viral addition. Each point is the average of 3 biological replicates, black bars indicate standard error. Red squares and curve: infected culture, blue squares and curve: healthy culture (**B**) aspect of an infected (left) and healthy (right) *T. striata* culture, 5 days after viral addition. Other biological replicates had the same aspect.

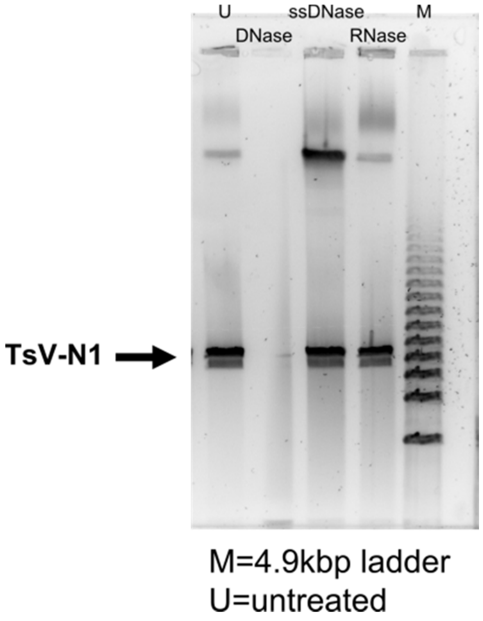

Figure S2. Genome characterization of the TsV-N1 viral isolates based on enzymatic digestions. Picture shows PFGE of viral genomes treated with dsDNase, ssDNase, and RNase. U—control sample, without enzyme treatment; M—marker ladder.

References

1. Sogin, M.L.; Gunderson, J.H. Structural diversity of eukaryotic small subunit ribosomal RNAsa. *Ann. N. Y. Acad. Sci.* **1987**, *503*, 125–139.
2. Amann, R.I.; Binder, B.J.; Olson, R.J.; Chisholm, S.W.; Devereux, R.; Stahl, D.A. Combination of 16S rRNA-targeted oligonucleotide probes with flow cytometry for analyzing mixed microbial populations. *Appl. Environ. Microbiol*. **1990**, *56*, 1919–1925.
3. Edgar, R.C. MUSCLE: Multiple sequence alignment with high accuracy and high throughput. *Nucleic Acids Res.* **2004**, *32*, 1792–1797.
4. Darriba, D.; Taboada, G.L.; Doallo, R.; Posada, D. jModelTest 2: More models, new heuristics and parallel computing. *Nat. Methods* **2012**, *9*, 772–772.
5. Guindon, S.; Dufayard, J.F.; Lefort, V.; Anisimova, M.; Hordijk, W.; Gascuel, O. New algorithms and methods to estimate maximum-likelihood phylogenies: Assessing the performance of PhyML 3.0. *Syst. Biol.* **2010**, *59*, 307–321.
6. Ronquist, F.; Teslenko, M.; van der Mark, P.; Ayres, D.L.; Darling, A.; Hohna, S.; Larget, B.; Liu, L.; Suchard, M.A.; Huelsenbeck, J.P. MrBayes 3.2: Efficient Bayesian phylogenetic inference and model choice across a large model space. *Syst. Biol.* **2012**, *61*, 539–542.
7. Rambaut, A.; Suchard, M.A.; Xie, D.; Drummond, A.J. Tracer v1.6. http://beast.bio.ed.ac.uk/Tracer. (accessed on 16 July 2015).
8. Sukumaran, J.; Holder, M.T.; DendroPy: A Python library for phylogenetic computing. *Bioinformatics* **2010**, *26*, 1569–1571.
9. Rozen, S.; Skaletsky, H. Primer3 on the www for general users and for biologist programmers. *Methods Mol. Biol.* **2000**, 132, 365–386.

© 2015 by the authors; licensee MDPI, Basel, Switzerland. This article is an open access article distributed under the terms and conditions of the Creative Commons Attribution license (http://creativecommons.org/licenses/by/4.0/).
